# Dynamics of Information Flow between the Chinese A-Share Market and the U.S. Stock Market: From the 2008 Crisis to the COVID-19 Pandemic Period

**DOI:** 10.3390/e24081102

**Published:** 2022-08-10

**Authors:** Chun-Xiao Nie, Jing Xiao

**Affiliations:** 1 School of Statistics and Mathematics, Zhejiang Gongshang University, Hangzhou 310018, China; 2 Collaborative Innovation Center of Statistical Data Engineering, Technology & Application, Zhejiang Gongshang University, Hangzhou 310018, China

**Keywords:** stock market, transfer entropy, information flow, surrogates

## Abstract

The relationship between the Chinese market and the US market is widely concerned by researchers and investors. This paper uses transfer entropy and local random permutation (LRP) surrogates to detect the information flow dynamics between two markets. We provide a detailed analysis of the relationship between the two markets using long-term daily and weekly data. Calculations show that there is an asymmetric information flow between the two markets, in which the US market significantly affects the Chinese market. Dynamic analysis based on weekly data shows that the information flow evolves, and includes three significant periods between 2004 and 2021. We also used daily data to analyze the dynamics of information flow in detail over the three periods and found that changes in the intensity of information flow were accompanied by major events affecting the market, such as the 2008 financial crisis and the COVID-19 pandemic period. In particular, we analyzed the impact of the S&P500 index on different industry indices in the Chinese market and found that the dynamics of information flow exhibit multiple patterns. This study reveals the complex information flow between two markets from the perspective of nonlinear dynamics, thereby helping to analyze the impact of major events and providing quantitative analysis tools for investment practice.

## 1. Introduction

Both China and the United States are large economies with high influence, so analyzing the relationship between the two stock markets is of great significance for investment practice and risk management. Currently, there are many studies analyzing the relationship between the two markets based on different datasets, in which multiple empirical tools are used. For example, Goh et al. found that some economic variables in the United States can improve the forecast performance of the Chinese stock market [1]. Jian Chen et al. found that US economic variables can help predict the monthly volatility of the Chinese market [2]. In addition, previous studies have also shown that US economic policy uncertainty can significantly explain the returns of China’s A-share market [3]. Researchers not only pay attention to how economic variables and policies in the US market affect the Chinese market but also directly discuss the relationship between the returns of the two markets. These studies focus on the impact of special events on the relationship between markets, such as the 2007–2009 crisis [4,5,6,7,8,9,10], the Eurozone crisis [11], and the COVID-19 pandemic [12,13,14]. Empirical analysis shows that the financial crisis has had a significant impact on the BRICS and emerging markets, such as leading to significant risk spillover effects and risk contagion [5,6,7]. In particular, the Chinese market was significantly affected by the US market during the crisis [4,5,6,8,10,11]. These studies used many types of quantitative tools, such as multivariate GARCH models [4,6,10], Granger causality test [5], latent factor models [9], correlation contagion tests [8], etc. In particular, recently, the COVID-19 pandemic has significantly impacted the Chinese market and the US market [12]. For example, research reveals that China’s stock market and the US stock market have significant spillover effects during the pandemic [13]. In addition, the pandemic has also impacted the correlation structure between market indexes [14].

Existing studies have revealed that there are extensive links between markets, and the Chinese market is significantly affected by the US market, especially under the impact of major events. However, previous studies have not fully analyzed the long-term relationship between the two markets. For example, most studies only analyze the static relationship in a specified period but do not focus on dynamics. This study analyzes the long-term relationship between the two markets from the perspective of nonlinear information flow dynamics. We use transfer entropy (TE) to quantitatively characterize nonlinear relationships [15], and analyze the dynamics in detail by surrogate time series [16]. Surrogates is an effective technique for analyzing nonlinear time series, which generates simulated time series by keeping some characteristics of the original time series [17,18,19].

At present, transfer entropy has expanded into a “toolbox” that includes multiple tools. For example, Marschinski et al. introduced effective transfer entropy by shuffling the source time series, where the shuffling process is used to destroy the correlation [20]. Further, the group transfer entropy and the effective group transfer entropy can be constructed by considering multiple time series [21]. Jizba et al. constructed the Rényian transfer entropies by extending Shannon entropy to Rényi entropy [22]. Papana et al. extended the transfer entropy method to analyze non-stationary time series [23]. Recently, Nie proposed a method to detect local information flow [16]. This method obtains the baseline TE value from local surrogates so that it is possible to observe the contribution of observations within a small computational window to the original TE value. An advantage of this method is that changes in the intensity of the information flow can be clearly observed, so that we can identify the impact of events on the information flow [16].

Since transfer entropy is a non-parametric method that effectively extracts the causal relationship between variables, it is suitable for analyzing high-complexity financial time series. For example, methods based on transfer entropy have been widely used to analyze financial time series, such as the relationship between stock markets [24,25,26,27,28], the price—volume relationship [16,29,30,31], and the foreign exchange market [32]. In particular, early empirical research shows that the US market is a core source of information for global financial markets [28]. Kim et al. considered 10 important indexes and found that the structure of the information transfer network was affected by the crisis [33].

This study focuses on analyzing the evolutionary characteristics of the information flow between China’s A-share market and the US market. We use nonlinear analysis methods to characterize the complex information flow dynamics between the two markets and, in particular, identify periods of high information flow intensity by TE and LRP. This study reveals the details of information transfer between markets based on long-term data and from multiple time scales. In the following, we first describe the data set and analyze basic descriptive statistics. Secondly, we review the concept and calculation method of transfer entropy, and the steps of LRP-based analysis. Third, we use weekly data and daily data to analyze the information flow between the two markets. Finally, we analyze the information flow between the S&P500 index and the industry indices of the Chinese A-share market.

## 2. Data

This paper uses three datasets, which include closing indices for two market composite indices and some industry indices. The composite index data includes daily and weekly closing data of S&P500 and CSI300 indices, as well as weekly closing data of some industry indices in the Chinese A-share market. All data are extracted from the iFinD database of Tonghuashun company. For the original closing index time series P={P(t)}, we calculate the series R={R(t)}, where R(t)=log(P(t+1))−log(P(t)).

For weekly data, we use $R_{us}$ and $R_{cn}$ to represent the return series of the S&P500 index and the CSI300 Index, respectively. We considered the data from 31 December 2004 to 17 June 2022, a total of 890 observations, so that each preprocessed series includes 889 observations. Table 1 lists some descriptive statistics of the two series. In addition, the Pearson correlation coefficient of the two-time series is 0.2118, which implies a weak correlation.

In addition to the weekly data, we also analyzed the daily data of the two indexes. To clearly analyze the impact of major events, we divide the daily data into three periods. For the preprocessed index data, we use $R_{cn}^{1}$ and $R_{us}^{1}$ to represent the data of the first period and use similar symbols for other periods. The time intervals corresponding to the three data sets are 4 January 2007–31 December 2010, 2 January 2014–29 December 2017, and 2 January 2018–31 December 2021 respectively. Since the trading days of the two markets are not synchronized, we remove the missing values in the two series, leaving observations with the same time stamp. The time series of the three periods without missing values include 944, 948, and 942 observations, respectively. Table 1 lists the basic descriptive statistics of each return series. Here, we always use the rounding method to keep four decimal places.

We can find from Table 1 that all the averages are close to zero. In three of the four pairs of time series, the standard deviation of the series in the Chinese market is greater than that in the US market. In addition, all skewness values are less than zero, and in particular, all excess kurtosis values are greater than zero. Here, we report the excess kurtosis obtained by subtracting 3 from the original kurtosis value. The kurtosis values imply that all distributions have peak characteristics and are not normal.

In addition to the market composite index, we used 31 industry indices compiled by SWS Research Co., Ltd. Here, we used three of them for dynamic analysis. Table 1 lists the descriptive statistics of the return series for the three indices. The symbols $R_{ind}^{1}$, $R_{ind}^{2}$ and $R_{ind}^{3}$ correspond to the industry index codes, 801040.SL (Steel industry), 801180.SL (Real estate) and 801960.SL (Petrochemical industry), respectively. It can be found that the average of $R_{ind}^{2}$ is significantly larger than the other two indices, while the difference between the standard deviations is small. In particular, all skewness values are less than 0, and kurtosis values are greater than 0.

## 3. Method

### 3.1. Transfer Entropy

We consider two random variables *I* and *J*, the marginal probability distribution is $p_{I}\left(\cdot \right)$ is $p_{J}\left(\cdot \right)$, and the joint distribution is $p_{I,J}\left(\cdot \right)$. We assume that the underlying dynamic structure conforms to a stationary Markov process, where *I* and *J* correspond to orders *k* and *l*, respectively. For *I*, the conditional distribution of state at time t+1 ($i_{t+1}$) is independent of state in t−k ($i_{t-k}$), that is, $p_{I}\left(i_{t+1}|i_{t},\cdots ,i_{n-k+1}\right)=p_{I}\left(i_{t+1}|i_{t},\cdots ,i_{n-k}\right)$. Similarly, for *J*, $p_{J}\left(j_{t+1}|j_{t},\cdots ,j_{n-k+1}\right)=p_{J}\left(j_{t+1}|j_{t},\cdots ,j_{n-k}\right)$, where $j_{t}$ represents the state at time *t*. The transfer entropy of J→I is defined as Equation (1) [15], where the symbols $i_{t}^{k}=\left(i_{t},\cdots ,i_{n-k+1}\right)$ and $j_{t}^{k}=\left(j_{t},\cdots ,j_{n-k+1}\right)$. In this article, we set l=k=1.

(1) $$TE_{J\to I}=\sum p\left(i_{t+1},i_{t}^{k},j_{t}^{l}\right)log\frac{p(i_{t+1}|i_{t}^{k},j_{t}^{l})}{p(i_{t+1}|i_{t}^{k})}$$

### 3.2. The Method Used to Estimate the Transfer Entropy

For a time series, we can encode it into a time series including states and then estimate the transfer entropy. Here, we use a method proposed in previous studies to encode the time series [25,26,34], which encodes the original time series through a group of quantiles. One of the advantages of this method is that a large weight can be assigned to the tail of the distribution, thereby focusing on the influence of extreme values in the financial market [25,26]. In addition, we use the R package RTransferEntropy developed by Behrendt et al. [34].

If the time series s={s(t)} is coded into *n* states, n−1 quantiles $Q=\{q_{1},q_{2},\cdots ,q_{n-1}\}$ are required, as shown in Equation (2). For example, if the observation s(t) is between the quantiles $q_{2}$ and $q_{3}$ of the distribution, then $s^{\prime }\left(t\right)=2$. The converted time series $s^{\prime }=\left\{s^{\prime }\left(t\right)\right\}$ includes *n* states, and can be used for conditional distribution and joint distribution.

(2) $$s^{\prime }\left(t\right)=\left\{\begin{array}{ccc} 1 & \; & s\left(t\right)\leq q_{1}\\ 2 & \; & s\left(t\right)\in \left(q_{2},q_{3}\right)\\ \cdots \\ n-1 & \; & s\left(t\right)\in \left(q_{n-2},q_{n-1}\right)\\ n & \; & s\left(t\right)\geq q_{n-1} \end{array}\right.$$

In order to comprehensively analyze the significance of information flow at different quantile parameters, we set the following five sets of parameters: $Q_{1}=\{q\left(0.05\right),q\left(0.5\right),$q(0.95)}, $Q_{2}=\left\{q\left(0.10\right),q\left(0.5\right),q\left(0.90\right)\right\}$, $Q_{3}=\{q\left(0.15\right),q\left(0.5\right),$q(0.85)}, $Q_{4}=\{q\left(0.20\right),$q(0.5),q(0.80)} and $Q_{5}=\{q\left(0.25\right)$,q(0.5),q(0.75)}, where the symbol q(α) represents α-quantile. From the first to the fifth set of parameters, the weight of the extreme values of the tail decreases. In the first group, extreme values are given the greatest weight, and the distribution in the fifth group is equally divided into four parts.

Here, we use the method proposed by Dimpfl et al. to test the significance of TE [25,34]. The method first estimates the dynamics between two variables through the Markov process. Secondly, based on the Markov process, a pair of simulated time series whose dependence has been eliminated can be generated, and the TE value can be estimated. Third, we repeatedly generate the simulated time series $N_{boot}$ times to obtain a TE value distribution for comparison. Finally, the *p*-value is generated by comparing the original TE value with the quantile of the baseline distribution. In this article, we always set $N_{boot}=1000$.

### 3.3. LRP-Based Analysis

The local random permutation (LRP) method uses RP surrogates locally to estimate the intensity of the information flow within a period [16]. Previous studies have confirmed that this method can effectively describe changes in information flow through toy models [16]. In particular, to maintain the correlation between series, LRP provides surrogates that keep the Pearson correlation unchanged.

We consider the information flow $s_{1}\to s_{2}$ between the series $s_{1}=\left\{s_{1}\left(t\right)\right\}$ and $s_{2}=\left\{s_{2}\left(t\right)\right\}$, where each series includes *N* observations. We assume that the length of the calculation window for constructing local RP surrogates is $L_{w}$, and the $TE_{s_{1}\to s_{2}}$ value is significant.

1 We extract the observations in the time interval $\left[1,L_{w}\right]$ to obtain the sub-time series $s_{1}^{\left[1,L_{w}\right]}=\left\{s_{1}\left(1\right),\cdots ,s_{1}\left(L_{w}\right)\right\}$ and $s_{2}^{\left[1,L_{w}\right]}=\left\{s_{2}\left(1\right),\cdots ,s_{2}\left(L_{w}\right)\right\}$, and shuffle the observations of the sub-sequences $s_{1}^{\left[1,L_{w}\right]}$ and $s_{2}^{\left[1,L_{w}\right]}$. In the scrambling step, the correspondence between the timestamps of the two series does not change, that is, the series $\left\{\left(s_{1}\left(k\right),s_{2}\left(k\right)\right)\right\}$ composed of pairs is scrambled.
2 We calculate the transfer entropy value between the time series obtained by local scrambling in step 1, and denoted it as $TE_{s_{1}\to s_{2}}^{\left[1,L_{w}\right]}\left(1\right)$.
3 We repeat steps 1 and 2 a total of *M* times to obtain a distribution of TE values used as a benchmark ($TE_{ben}^{1}=\left\{TE_{s_{1}\to s_{2}}^{\left[1,L_{w}\right]}\left(k\right)|k=1,2,\cdots ,M\right\}$).
4 We calculate the *Z*-score $Z_{\left[1,L_{w}\right]}=\frac{TE-m_{\left[1,L_{w}\right]}}{s_{\left[1,L_{w}\right]}}$.
5 We move the calculation window one observation each time, and repeat steps 1–4 to get the distribution $TE_{ben}^{i}=\left\{TE_{s_{1}\to s_{2}}^{\left[i,L_{w}\right]}\left(k\right)|k=1,2,\cdots ,M\right\}$ and the *Z*-score. For example, the second *Z*-value is obtained by scrambling all observations in the interval $\left[2,L_{w}+1\right]$. Finally, we get the *Z*-score sequence $\left\{Z_{\left[i,i+L_{w}-1\right]}|i=1,2,\cdots ,N-L_{w}+1\right\}$.

The estimation of the TE value requires setting the parameter $Q_{k}$, so the parameter may affect the calculation result. Here, we use the following method to synthesize the calculation results of different parameters. For a sequence of *Z*-scores ($\left\{z_{k}\left(t\right)\right\}$) generated by a parameter $Q_{k}$, we construct a 0–1 sequence using Equation (3), where $Z_{th}$ is the threshold. We set the threshold $Z_{th}=1.645$ here. In this way, we obtain a series $I_{k}=\left\{I_{k}\left(t\right)|t=1,2,\cdots ,N-L_{w}+1\right\}$ based on *Z*-scores, in which observations only take values in {0,1}. If $TE_{ben}^{i}$ is a normal distribution, then the original TE value is greater than most of the values (95%) in $TE_{ben}^{i}$, which implies that there is a significant information flow in the period $\left[i,i+L_{w}-1\right]$. In this article, we always set M=1000. Then, we calculate the series I={I(t)} ($I\left(t\right)=\sum_{k}I_{k}\left(t\right)$).

(3) $$I_{k}\left(t\right)=\left\{\begin{array}{ccc} 1, & \; & z_{k}\left(t\right)\geq z_{th}\\ 0, & \; & z_{k}\left(t\right)<z_{th} \end{array}\right.$$

Since each *Z*-score corresponds to an interval, in this article, we use the following convention to plot the figure: the right end of the interval corresponding to the value is used as the time stamp in the figure. For example, for the value $Z_{\left[1,L_{w}\right]}$, the time label in the figure is the timestamp corresponding to $L_{w}$. Similarly, we plot the time series {I(t)}.

## 4. Results

### 4.1. Information Flow Analysis Based on Weekly Data

#### 4.1.1. TE Values of Different Parameters

We use a group of parameters $Q_{k}$ (k=1,⋯,5) to calculate the information flow between $R_{us}$ and $R_{cn}$. Table 2 shows the calculated results, all $TE_{us\to cn}$ values are significant at the significance level α=0.05, and three of the *p*-values are less than 0.01. In addition, all $TE_{cn\to us}$ values are not significant. Table 2 suggests that there is an asymmetric information flow between the Chinese market and the US market, and the US market is a dominant information source.

The minimum *p*-value of 0.005 corresponds to the parameter $Q_{2}$, which means that significant information flow can be detected when a large weight is assigned to extreme values. In addition, the *p*-value corresponding to the equally divided quantile interval is also less than 0.05. This means that significant us→cn information flow can be detected regardless of whether a greater weight is given to the extreme values.

In summary, there is sufficient evidence to support the existence of information flow us→cn. The change of the *p*-value with the change of the parameter implies that the analysis of the information flow depends on the choice of the parameter, and thus the calculation results of multiple parameters need to be integrated.

#### 4.1.2. Information Flow Dynamics for Weekly Data

In the previous section, we analyzed the information flow between the two markets globally. However, the static analysis only shows the existence of information flow in the considered period, thus lacking local dynamic analysis. Below, we use LRP to analyze the dynamics of information flow, in particular, to detect periods with localized strong information flow.

**Figure 1:**
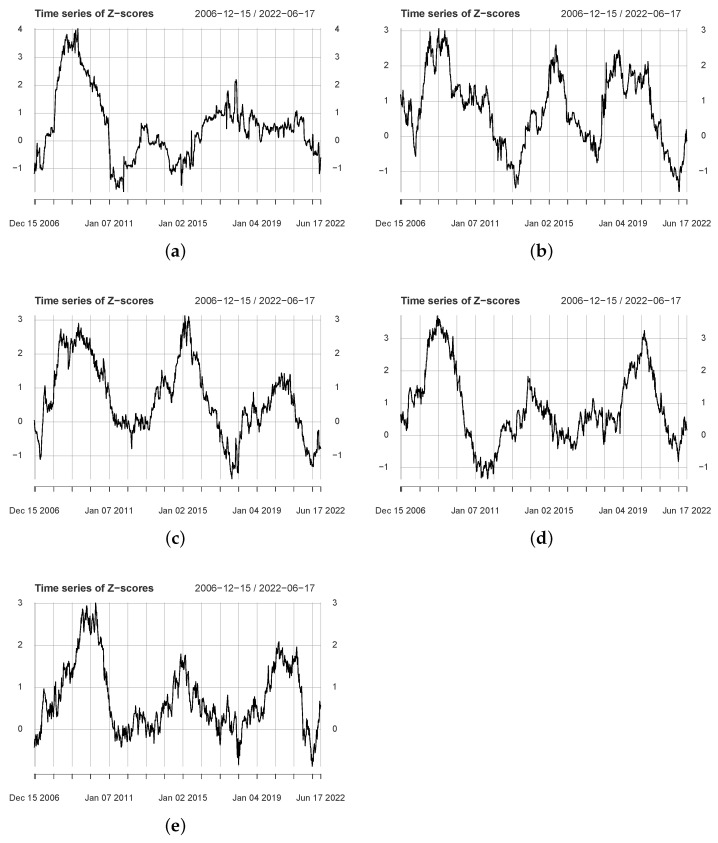
The figure shows the *Z*-scores for different parameters *Q*, where (**a**–**e**) correspond to $Q_{1}$–$Q_{5}$, respectively.

We set five sets of parameters $Q_{k}$ (k=1,⋯,5) and $L_{w}=96$, where the calculation window corresponds to two years of trading weeks. The five subfigures of Figure 1 show the *Z*-score time series. We observe that all subfigures include local peaks larger than 1.645, implying non-trivial information flow dynamics between series. These peaks correspond to periods of localized strong information flow. For example, for Figure 1a, it can be seen that the *Z*-score increased rapidly in 2008 and dropped significantly thereafter. Furthermore, we can also observe that the number of peaks in all *Z*-score series is greater than 1. For example, three localized peaks can be observed in Figure 1b, implying the existence of three periods of strong information flow.

The numbers of local peaks for the five *Z*-score series are 2, 3, 2, 3, and 3, respectively. Table 3 lists the periods corresponding to these peaks. We use $T_{1}$, $T_{2}$, and $T_{3}$ to represent the time intervals in three periods, where each period corresponds to two endpoints. For example, the starting time corresponding to $T_{1}$ ($Q_{1}$) is 8 June 2007, and the closing time is 24 April 2009. The first line indicates that there is a significant flow of information in the period 8 June 2007–24 April 2009. We list the *Z*-score at the corresponding time interval in the fourth column. The maximum value corresponds to the $T_{1}$ period, while the minimum value corresponds to the $T_{2}$ period.

We find that most of the local peaks correspond to periods that are close to each other, such as the $T_{1}$ period of parameter $Q_{1}$ and the $T_{1}$ period of $Q_{2}$ are 8 June 2007–24 April 2009 and 2 March 2007–19 January 2009 respectively. Table 3 shows that the 2008 crisis significantly affected the information flow of us→cn. In addition, the periods corresponding to several local peaks also include major events. For example, the third peak of $Q_{5}$ includes the oil crisis and the collapse of the US stock market. In summary, the LRP-based method identifies some local peaks, indicating that the dynamics of information flow are non-trivial.

#### 4.1.3. Comprehensive Analysis of Z-Score Series

We roughly identified three important periods by *Z*-scores in the previous subsection. Below, we calculate the *I* series from the $I_{k}$ series of five *Z*-score series. The series *I* takes the value in the set {0,1,2,3,4,5}. If $I_{k}\left(t\right)=0$, the observations in the time period $\left[t-L_{w}+1,t\right]$ do not significantly affect the analysis results of the information flow. On the contrary, if I(t)=5, it implies that the information flow within the period $\left[t-L_{w}+1,t\right]$ is significant and does not depend on the choice of parameters. A large I(t) value implies that there is information flow in multiple quantile intervals $\left\{Q_{k}|k=1,\cdots ,5.\right\}$.

**Figure 2:**
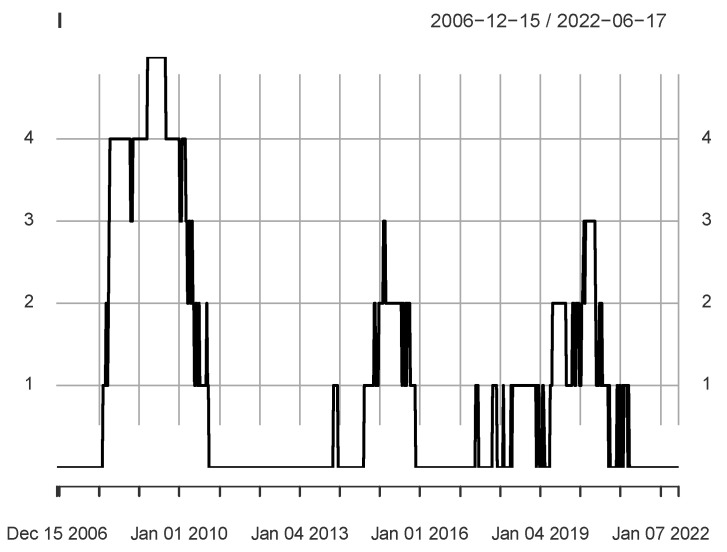
The *I* time series obtained from five sets of parameters, and takes values in {0,1,2,3,4,5}. It includes three local peaks, and the maximum corresponds to the 2008 crisis.

Figure 2 shows the *I* series. There are three periods where most observations are greater than or equal to 1. In particular, the analysis shows that there is a period where most observations are greater than or equal to 3, that is, 8 February 2008–24 September 2010. During the 2008–2009 crisis in the US market, there was strong information flow us→cn. We use $N_{i}$ to represent the number of observations equal to *i* in the *I* series, as shown in Table 4. It can be found that if the calculation results of 5 parameters are integrated, nearly half of the observations are greater than or equal to 1. This implies that information flows widely exist between the two markets during the period under consideration. Table 5 lists the three periods identified by Figure 2. Each period includes some major events that affect the market. The first period corresponds to the 2007–2009 crisis in the US market. The second period includes the crisis in the Chinese stock market in 2015. In addition, before the stock market crash in 2015, the bubble was generated in the second half of 2014. The second period not only includes the breakage period of the bubble, but also the generation period. The third period includes a series of major events, such as the circuit breaker in the US market in March 2020, and the COVID-19 pandemic. Based on this table, we divide three periods for the analysis of daily data in the next section: 2007–2010, 2014–2017, and 2008–2021.

### 4.2. Information Flow Analysis of Daily Data

#### 4.2.1. Global Analysis of Daily Data

In this section, we analyze the relationship between the daily data of the two indices. Table 6 lists the transfer entropy values $TE_{us\to cn}$ of different parameters. For the first and third periods, all TE values are significant at significance level α=0.05. For the second period, the TE values of parameters $Q_{1}$ and $Q_{2}$ were not significant. However, the *p*-value for the TE value of $Q_{4}$ was less than 0.01. This implies that extreme values have little effect on information flow in the second period. In summary, we still found significant information flows in the three periods of daily data. Below, we use LRP-based analysis to discuss the dynamics of information flow in each period.

In the next subsection, for the first period and the second period, we choose the parameter *Q* corresponding to the maximum TE value in Table 6. In the analysis of the first and third periods, the parameter *Q* was set to $Q_{4}$ and $Q_{2}$, respectively. In addition, the second period is a special period, in which both $Q_{1}$ and $Q_{2}$ correspond to insignificant TE values, suggesting that extreme values in this period weakly affect the level of information flow. For the second period, we set $Q_{3}$ and $Q_{4}$ for calculation.

#### 4.2.2. Information Flow Dynamics for 2007–2010 Data

**Figure 3:**
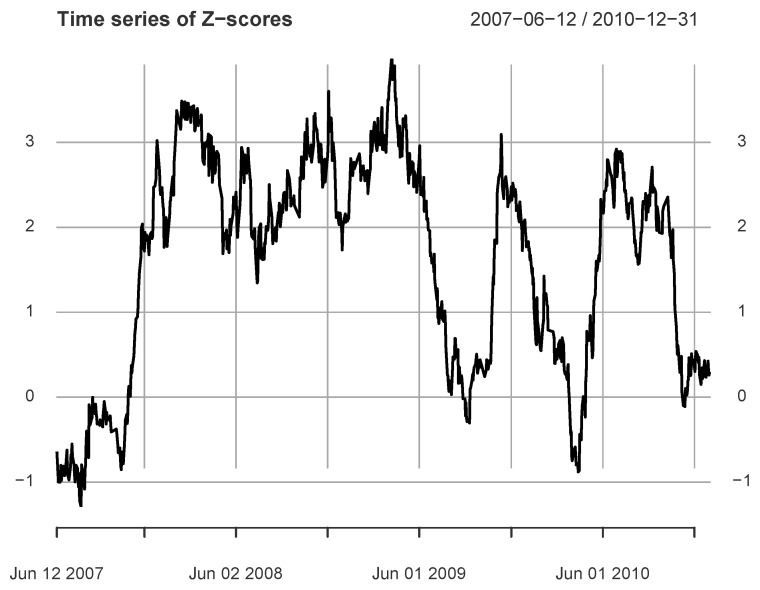
The figure shows the *Z*-score time series between $R_{us}^{1}$ and $R_{cn}^{1}$, where $L_{w}=100$.

Figure 3 shows the *Z*-score series for the 2007–2010 data. If the threshold is 1.645, the series includes three significant periods: 26 November 2007 (28 June 2007)–25 June 2009, 28 October 2009 (1 June 2009)–8 January 2010, and 24 May 2010 (21 December 2009)–19 October 2010. Here, the date in parentheses is the left endpoint of the calculation window for the first *Z*-score greater than 1.645 within the period. In particular, the maximum value (3.9880) is in the first period and the calculation window is 3 November 2008–7 April 2009.

The calculation results clearly show that the 2007–2009 crisis in the US market significantly affected the Chinese market. In addition, we also observe that the us→cn information flow also exists in the post-crisis period. These results imply not one peak with strong information flow, but multiple peaks with different intensities.

#### 4.2.3. Information Flow Dynamics for 2014–2017 Data

**Figure 4:**
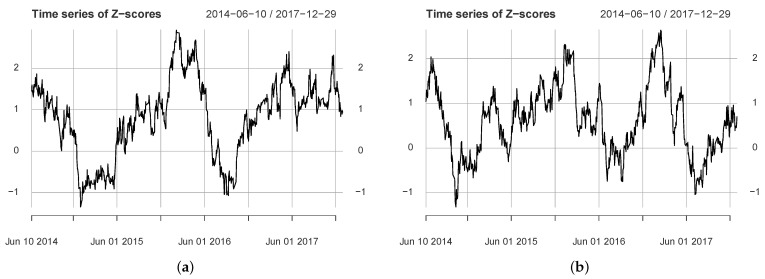
The figure shows the *Z*-score time series between $R_{us}^{2}$ and $R_{cn}^{2}$, where (**a**,**b**) correspond to parameters $Q_{3}$ and $Q_{4}$, respectively.

Figure 4 shows the information flow dynamics for the 2014–2017 data. Figure 4a includes a major significant period: 6 January 2016 (6 August 2015)–10 May 2016. The results show that the information flow in the us→cn direction is enhanced after the Chinese market bubble burst. Figure 4b shows the calculation results corresponding to $Q_{4}$. We found that it also includes a major significant period: 30 December 2016 (1 August 2016)–1 March 2017. The period included an important event that affected US markets: the 2016 presidential election. A previous study showed that the event significantly affected correlation dynamics in the US stock market [35]. The analysis here suggests that there was a significant information flow from the US stock market to the Chinese stock market around this event. The two results in Figure 4 show that from 2 January 2014 to 29 December 2017, the US market significantly influenced the Chinese market in two periods, and both periods included major events.

#### 4.2.4. Information Flow Dynamics for 2018–2021 Data

**Figure 5:**
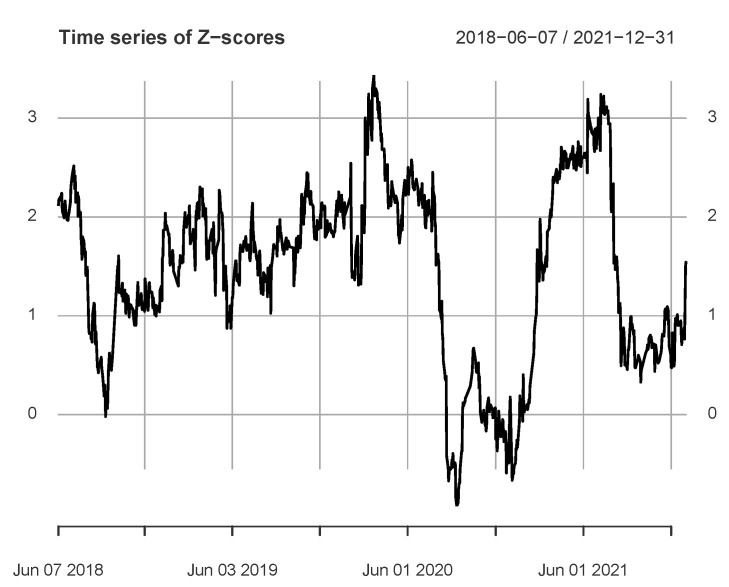
The figure shows the *Z*-score series for $R_{us}^{3}$ and $R_{cn}^{3}$, which includes two main peaks ($Q=Q_{2}$, $L_{w}=100$).

Figure 5 shows the calculation results for the 2018–2021 data. The period includes several sub-periods greater than the critical *Z*-score, suggesting that from 2 January 2018 to 31 December 2021, the flow of information between the two markets was active and strong. These sub-periods are as follows: 7 June 2018 (3 January 2018)–31 July 2018 ($T_{1}$), 22 February 2019 (12 September 2018)–15 May 2019 ($T_{2}$), 19 June 2019 (11 January 2019)–30 July 2019 ($T_{3}$), 27 August 2019 (29 March 2019)–5 February 2020 ($T_{4}$), 27 February 2020 (19 September 2019)–29 July 2020 ($T_{5}$), 3 March 2021 (24 September 2020)–2 August 2021 ($T_{6}$). We label the different subperiods with $T_{i}$. It can be found that $T_{2}$, $T_{3}$, $T_{4}$ and $T_{5}$ can be combined into an important period. There were several major events in this period: Sino-US trade friction, the oil crisis, the COVID-19 pandemic, the US stock market crisis in March 2020. In addition, periods $T_{1}$ and $T_{6}$ still belong to the period of Sino-US trade friction and the period of the COVID-19 pandemic, respectively. In particular, the calculation window corresponding to the maximum value (3.4339) is 21 October 2019–23 March 2020. Several major events are included in this period, such as the oil crisis, the COVID-19 pandemic, and the March 2020 market crisis. To sum up, it is reasonable to speculate that the impact of multiple major events led to the active information flow during the period.

### 4.3. Information Flow between Industry Indices in the Chinese Market and the S&P500 Index

#### 4.3.1. Global Information Flow Analysis between Industry and Market Index

Since the composite index includes stocks belonging to various industries, in this section, we analyze the relationship between the S&P500 index and the industry index in the Chinese market. Here, we consider the case where a larger weight is given to the tail of the distribution, i.e., $Q=Q_{1}$. Table 7 lists the TE values and *p*-values, where the symbol “ind” represents the industry index, and the code is listed in the second column.

The fourth and sixth columns show that there are several industry indices that are closely related to the S&P500 index. For example, there is a significant bidirectional flow of information between the petrochemical industry and the S&P500 index. In addition, some unidirectional effects can also be found, such as in real estate. It should be noted that not all industry indices are affected by the S&P500 index, such as the public utilities index and the architectural ornament index. To sum up, Table 7 shows that the information flow from S&P500 to Chinese market industry indices is heterogeneous.

#### 4.3.2. The Dynamics of Information Flow between Industry Index and S&P500 Index

In the previous section, we found diversity in the relationship between the S&P500 index and industry indices, with some industry indices having a stronger flow of information between them and the S&P500 index. A related issue is whether the dynamics of information flow for different industry indices have similar patterns. Here, we select three industry indices and compare information flow dynamics. The three indices are 801040.SL (Steel industry), 801180.SL (Real estate) and 801960.SL (Petrochemical industry), and all have significant us→ind information flow.

**Figure 6:**
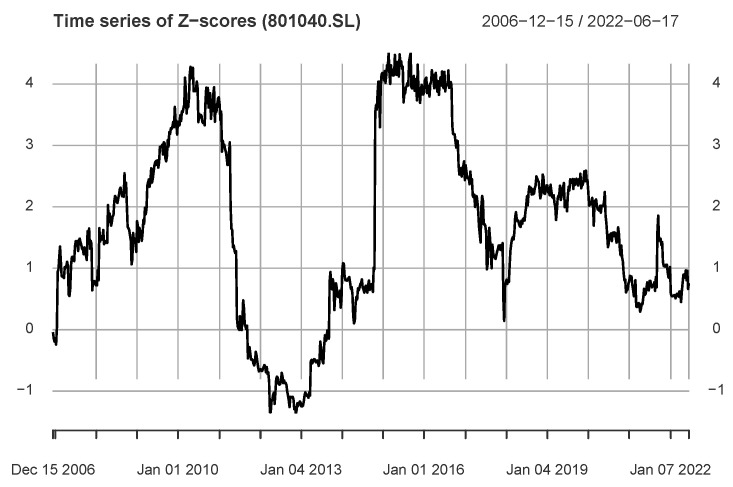
The figure shows the *Z*-score time series for the industry index 801040.SL (Steel industry) and the S&P500 index, including two clear local peaks.

We analyze dynamics using weekly data and always set $Q=Q_{1}$ and $L_{w}=96$ weeks. Figure 6 shows the dynamics of information flow for us→801040.SL. The *Z*-score series includes 794 values, and 396 values are greater than the critical threshold, implying that there is a continuous flow of information us→801040.SL during the period. In addition, it includes several local peaks, and the corresponding periods of the two main peaks are 18 April 2008 (2 June 2006)–15 April 2011 and 24 October 2014 (21 December 2012)–30 June 2017, respectively.

**Figure 7:**
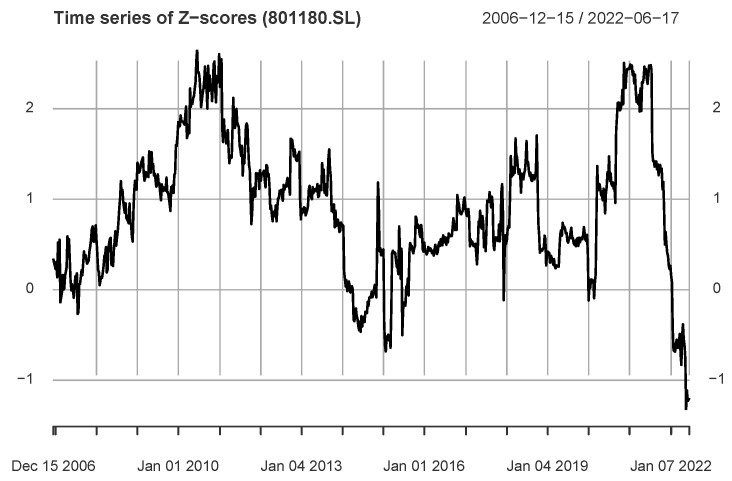
The figure shows the *Z*-score time series for the industry index 801180.SL (Real estate) and the S&P500 index, with only a few *Z*-score values above the critical threshold.

Figure 7 presents the *Z*-score series for the S&P500 Index → Real estate Index. For this *Z*-score series, there are only 124 values greater than the critical threshold. It shows a different pattern from Figure 6, such as observations in the period 4 September 2020 (26 October 2018)–16 July 2021 contribute significantly to the information flow. In addition, another major peak corresponds to the period 25 December 2009 (15 February 2008)–24 June 2011.

**Figure 8:**
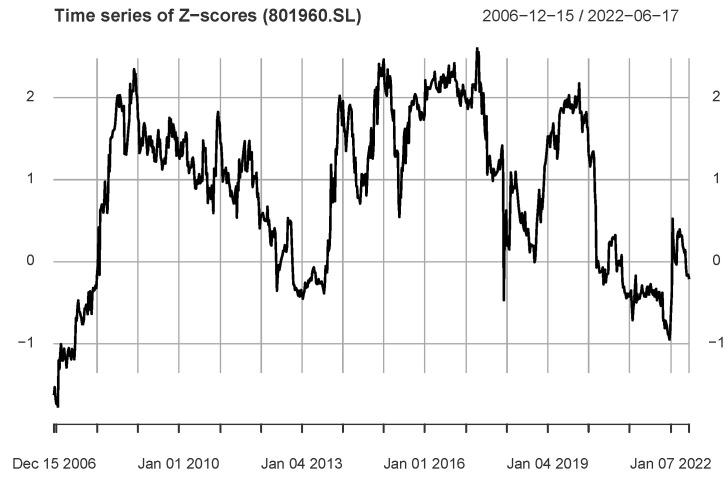
The figure shows the *Z*-score time series of the industry index 801960.SL (Petrochemical industry) and S&P500 index, and most of the Z-score values greater than the critical threshold are in the period 2014–2017.

The information flow analysis result of us→801960.SL is shown in Figure 8. We find that it included a period with most values greater than the critical threshold: 10 October 2014–9 June 2017. This period is close to that of the second peak in Figure 6. However, for the series shown in Figure 8, it includes some values less than the critical threshold.

The results in Figure 6, Figure 7 and Figure 8 show that the information flow dynamics of us→ind exhibit multiple patterns, thus indicating a high complexity of information flow between markets.

## 5. Discussion

This study examines periods of strong information flow between two important markets, where these periods are accompanied by major events. This implies that long-term major events may affect market relations more deeply. This provides a forward-looking analysis for analyzing the relationship between the two markets. In particular, we can analyze the dynamics of information flow in detail to identify changes in the relationship between markets when markets are impacted by major events. A related topic that can be studied is whether non-linear information flow helps predict future returns in the market.

Several previous studies have revealed that major events affect the relationship between markets [4,5,6,7,8,9,10,11,12,13,14]. Unlike previous studies, we analyze long-term data and focus on the dynamics of information flow. In particular, LRP-based analysis characterizes the strength of the local information flow, so that the time-varying characteristics of the information flow in a period can be observed in detail.

## 6. Conclusions

We discussed the long-term relationship between the Chinese market and the US market and found that there is an asymmetric information flow, where the US market is the source of information flow. Calculations show that the information flow between the Chinese market and the US market is not stable, but there are some significant periods. The LRP-based analysis identified three periods of significant information flow. All three periods include some major events affecting the market, such as the 2008 crisis, the oil crisis, and the COVID-19 pandemic, thus suggesting that the dynamics of information flow may be impacted by major events. In addition, we conducted a detailed dynamic analysis with daily data. Calculations show that each period includes nontrivial dynamics, some of which appear as local peaks, suggesting dramatic changes in information flow.

We also analyzed the information flow between the S&P500 index and some industry indices in the Chinese A-share market. Calculations show that the S&P500 index does not always significantly affect each industry index. Some industries are significantly affected, such as the steel industry and real estate. However, analysis based on weekly data shows that the dynamics of information flow between the industry indices and the S&P500 index exhibits multiple patterns. In addition, there are also some significant information flows from the Chinese market to the US market, thus demonstrating the heterogeneity of information flow.

This study demonstrates the high complexity of nonlinear causal relationships between two important markets and facilitates quantitative studies of the impact of major events. In particular, empirical analysis suggests that the heterogeneity of information flow in industry indices needs to be taken into account when discussing risk spillovers between markets. In this article, we only consider the information flow between the two markets. A related and important topic is the study of how major events affect information flow networks that include multiple markets. Since LRP-based methods involve two variables, for the information flow network, we need to construct a method that includes multiple variables. An alternative approach is to characterize changes in network structure by shuffling observations. In addition, the relationship between industry indices within a market can be analyzed in detail through LRP-based methods and high-frequency data. For example, for a trading day, we can analyze how important announcements impact the information flow network constructed by multiple industry indices.

## Figures and Tables

**Table 1:** Descriptive statistics of preprocessed time series.

Series	Average	Std	Skewness	Kurtosis
$R_{us}$	0.0012	0.0261	−2.0557	23.1000
$R_{cn}$	0.0016	0.0359	−0.2955	2.2400
$R_{us}^{1}$	$-1.2752\times 10^{-4}$	0.0179	−0.4871	9.0371
$R_{cn}^{1}$	$4.3938\times 10^{-4}$	0.0242	−0.5126	2.0974
$R_{us}^{2}$	$3.9919\times 10^{-4}$	0.0077	−0.2361	4.2776
$R_{cn}^{2}$	$5.8243\times 10^{-4}$	0.0161	−0.9794	6.7738
$R_{us}^{3}$	$6.0558\times 10^{-4}$	0.0138	−1.0405	17.2147
$R_{cn}^{3}$	$2.0142\times 10^{-4}$	0.0134	−0.3052	3.6399
$R_{ind}^{1}$	0.0007	0.0437	−0.2044	2.0224
$R_{ind}^{2}$	0.0016	0.0460	−0.3658	2.0825
$R_{ind}^{3}$	0.0007	0.0391	−0.3157	1.7477

**Table 2:** Information flow analysis of long-term weekly data.

$Q_{k}$	${\mathrm{TE}}_{\mathrm{cn}\to \mathrm{us}}$	$p_{\mathrm{cn}\to \mathrm{us}}$	${\mathrm{TE}}_{\mathrm{us}\to \mathrm{cn}}$	$p_{\mathrm{us}\to \mathrm{cn}}$
$Q_{1}$	0.040537	0.054	0.048419	0.006
$Q_{2}$	0.0352622	0.261	0.0519378	0.005
$Q_{3}$	0.0381743	0.141	0.0449418	0.039
$Q_{4}$	0.0357750	0.183	0.0492554	0.008
$Q_{5}$	0.047230	0.014	0.044303	0.031

**Table 3:** Three significant periods of the information flow of parameter.

*Q*	$T_{i}$	*t*	*Z*	Period
$Q_{1}$	$T_{1}$	24 April 2009	4.0550	8 June 2007–24 April 2009
$Q_{1}$	$T_{2}$	17 November 2017	2.2084	1 January 2016–17 November 2017
$Q_{2}$	$T_{1}$	9 January 2009	3.0700	2 March 2007–19 January 2009
$Q_{2}$	$T_{2}$	15 May 2015	2.5980	19 July 2013–15 May 2015
$Q_{2}$	$T_{3}$	12 October 2018	2.4534	2 December 2016–12 October 2018
$Q_{3}$	$T_{1}$	8 May 2009	2.9067	22 June 2007–8 May 2009
$Q_{3}$	$T_{2}$	17 April 2015	2.9681	21 June 2013–17 April 2015
$Q_{4}$	$T_{1}$	2 January 2009	3.6279	16 February 2007–2 January 2009
$Q_{4}$	$T_{2}$	8 November 2013	1.8349	23 December 2011–8 November 2013
$Q_{4}$	$T_{3}$	28 February 2020	3.2564	13 April 2018–28 February 2020
$Q_{5}$	$T_{1}$	9 April 2010	3.0202	23 May 2008–9 April 2010
$Q_{5}$	$T_{2}$	21 November 2014	1.8017	18 March 2013–21 November 2014
$Q_{5}$	$T_{3}$	13 March 2020	2.0941	27 April 2018–13 March 2020

**Table 4:** Statistics of *I* series.

$N_{i}$	$N_{1}$	$N_{2}$	$N_{3}$	$N_{4}$	$N_{5}$	Sum
Number	142	80	29	66	24	341

**Table 5:** Significant periods identified by Figure 2.

$T_{i}$	Period
$T_{1}$	8 February 2008–24 September 2010
$T_{2}$	15 August 2014–20 November 2015
$T_{3}$	13 April 2018–19 March 2021

**Table 6:** Analysis of TE value of daily data.

Period	2007–2010	2014–2017	2018–2021
$Q_{1}$	0.0573 (<0.001)	0.0322 (0.262)	0.0587 (<0.001)
$Q_{2}$	0.0490 (0.006)	0.0286 (0.529)	0.0630 (<0.001)
$Q_{3}$	0.0510 (0.002)	0.0505 (0.002)	0.0545 (<0.001)
$Q_{4}$	0.0601 (<0.001)	0.0489 (0.006)	0.0519 (<0.001)
$Q_{5}$	0.0600 (<0.001)	0.0407 (0.041)	0.0577 (<0.001)

**Table 7:** TE values of industry indices and S&P500 index.

Industry	Code	${\mathrm{TE}}_{\mathrm{ind}\to \mathrm{us}}$	$p_{\mathrm{ind}\to \mathrm{us}}$	${\mathrm{TE}}_{\mathrm{us}\to \mathrm{ind}}$	$p_{\mathrm{us}\to \mathrm{ind}}$
AFAF	801010.SL	0.0341	0.254	0.0444	0.011
Basic chemicals	801030.SL	0.0448	0.012	0.0394	0.061
Steel industry	801040.SL	0.0350	0.093	0.0686	0.000
Nonferrous metals	801050.SL	0.0353	0.125	0.0475	0.005
Electronics	801080.SL	0.0325	0.334	0.0413	0.034
Household Electric Appliance	801110.SL	0.0280	0.551	0.0622	0.000
Food and beverage	801120.SL	0.0293	0.491	0.0443	0.012
Textile, clothing.	801130.SL	0.0415	0.055	0.0483	0.004
Light manufacturing	801140.SL	0.0378	0.102	0.0504	0.000
Bio-pharmaceutical industry	801150.SL	0.0294	0.499	0.0418	0.036
Public utilities	801160.SL	0.0431	0.022	0.0359	0.121
Transportation industry	801170.SL	0.0338	0.257	0.0519	0.001
Real estate	801180.SL	0.0304	0.427	0.0469	0.008
Retail business	801200.SL	0.0388	0.079	0.0358	0.154
Social services	801210.SL	0.0407	0.040	0.0428	0.028
Comprehensive company	801230.SL	0.0296	0.482	0.0476	0.007
Building material	801710.SL	0.0347	0.213	0.0468	0.009
Architectural ornament	801720.SL	0.0393	0.072	0.0302	0.379
Power equipment	801730.SL	0.0279	0.559	0.0347	0.160
National defense industry	801740.SL	0.0342	0.239	0.0314	0.351
Computer industry	801750.SL	0.0253	0.719	0.0383	0.096
Media industry	801760.SL	0.0309	0.334	0.0484	0.006
Communication industry	801770.SL	0.0423	0.047	0.0414	0.031
Banking	801780.SL	0.0457	0.021	0.0333	0.220
Non-bank Financial Institutions	801790.SL	0.0251	0.775	0.0372	0.087
Automobile industry	801880.SL	0.0270	0.591	0.0508	0.004
Mechanical equipment	801890.SL	0.0443	0.021	0.0447	0.017
Coal industry	801950.SL	0.0418	0.031	0.0424	0.016
Petrochemical industry	801960.SL	0.0510	0.001	0.0469	0.004
Environmental protection	801970.SL	0.0282	0.595	0.0475	0.006
Beauty care	801980.SL	0.0337	0.258	0.0436	0.017

## Data Availability

Not applicable.
